# A subspace projection approach to quantify respiratory variations in the f-wave frequency trend

**DOI:** 10.3389/fphys.2022.976925

**Published:** 2022-09-19

**Authors:** Mostafa Abdollahpur, Gunnar Engström, Pyotr G. Platonov, Frida Sandberg

**Affiliations:** ^1^ Department of Biomedical Engineering, Lund University, Lund, Sweden; ^2^ Department of Clinical Sciences, Cardiovascular Research—Epidemiology, Malmö, Sweden; ^3^ Department of Cardiology, Clinical Sciences, Lund University, Lund, Sweden

**Keywords:** atrial fibrillation, autonomic nervous system, respiratory variation, f-wave frequency, ECG processing

## Abstract

**Background:** The autonomic nervous system (ANS) is known as a potent modulator of the initiation and perpetuation of atrial fibrillation (AF), hence information about ANS activity during AF may improve treatment strategy. Respiratory induced ANS variation in the f-waves of the ECG may provide such information.

**Objective:** This paper proposes a novel approach for improved estimation of such respiratory induced variations and investigates the impact of deep breathing on the f-wave frequency in AF patients.

**Methods:** A harmonic model is fitted to the f-wave signal to estimate a high-resolution f-wave frequency trend, and an orthogonal subspace projection approach is employed to quantify variations in the frequency trend that are linearly related to respiration using an ECG-derived respiration signal. The performance of the proposed approach is evaluated and compared to that of a previously proposed bandpass filtering approach using simulated f-wave signals. Further, the proposed approach is applied to analyze ECG data recorded for 5 min during baseline and 1 min deep breathing from 28 AF patients from the Swedish cardiopulmonary bioimage study (SCAPIS).

**Results:** The simulation results show that the estimates of respiratory variations obtained using the proposed approach are more accurate than estimates obtained using the previous approach. Results from the analysis of SCAPIS data show no significant differences between baseline and deep breathing in heart rate (75.5 ± 22.9 vs. 74 ± 22.3) bpm, atrial fibrillation rate (6.93 ± 1.18 vs. 6.94 ± 0.66) Hz and respiratory f-wave frequency variations (0.130 ± 0.042 vs. 0.130 ± 0.034) Hz. However, individual variations are large with changes in heart rate and atrial fibrillatory rate in response to deep breathing ranging from −9% to +5% and −8% to +6%, respectively and there is a weak correlation between changes in heart rate and changes in atrial fibrillatory rate (*r* = 0.38, *p* < 0.03).

**Conclusion:** Respiratory induced f-wave frequency variations were observed at baseline and during deep breathing. No significant changes in the magnitude of these variations in response to deep breathing was observed in the present study population.

## 1 Introduction

Atrial fibrillation is known as the most common heart arrhythmia and is a growing public health concern worldwide. Atrial fibrillation has been estimated to affect 10 million people in the United States by 2050 (Miyasaka et al., 2006) and 17.9 million in Europe by 2060, with more than half of these patients aged 80 years or older (Krijthe et al., 2013). Atrial fibrillation is associated with increased mortality and morbidity resulting from stroke and congestive heart failure, and increased hospitalization costs (Patel et al., 2014). Despite progression in AF treatment, including medications aimed at controlling heart rate, rhythm, or both, and ablative therapy, finding the most accurate therapy for an individual patient is still problematic (Crandall et al., 2009). Historically, research has shown that multiple etiological mechanisms, such as atrial fibrosis, ion-channel dysfunction, autonomic imbalance, and genetic background, likely drive the factors associated with the maintenance and progression of AF (Lip et al., 2010; Fabritz et al., 2016).

This study focuses on respiratory modulation in the atrial activity during AF. It is well established from a variety of studies that the refractory period of the atria during atrial fibrillation can be influenced by various underlying mechanisms, including pathological changes, electrophysiological dynamics, and an imbalanced autonomic tone (Waldo, 2003; Nitta et al., 2004; Saksena et al., 2005). The refractory period of the atria has been found to have linear correlation with f-waves frequency (Capucci et al., 1995). The frequency of the f-waves in the ECG, also referred to as the atrial fibrillatory rate (AFR) (Platonov et al., 2014), has previously been analyzed with respect to ANS induded changes during AF. For instance (Stridh et al., 2003; Holmqvist et al., 2005), have shown that variation in the f-wave frequency during controlled respiration can be linked to the parasympathetic activity. Östenson et al. studied changes in the f-wave frequency in response to changes in ANS tone induced by tilt-test in 40 patients with persistent AF, results showed f-wave frequency decreased during head-down tilt (HDT) compared to baseline and increased during head-up tilt (HUT) (Östenson et al., 2017). In a previous study, we investigated changes in f-wave frequency variations in response to controlled respiration (Abdollahpur et al., 2021). In a study population of eight pacemaker patients with permanent AF recorded at baseline, during controlled respiration, and during controlled respiration after injection of atropine. Briefly, a high-resolution f-wave frequency trend obtained using model-based approach was filtered using a narrow bandpass filter with center frequency corresponding to respiration rate and fixed bandwidth. The envelope of the filtered frequency trend served as an estimate of the magnitude of the respiratory variation; the results indicated that this magnitude was affected by parasympathetic regulation (Abdollahpur et al., 2021).

The present study addresses main weaknesses of our previous study. In contrast to the previous study, where the AF patients had pacemakers set at a fixed heart rate, the present study is based on AF patients without pacemaker whose heart rate varies over time. Such variations in heart rate may affect the ANS and hence the ANS induced variations in atrial electrical activity. Second, the previous approach to quantify respiratory variation in the f-wave frequency is sensitive to noise and cannot handle time-varying respiration rates. Hence, the objectives of the present study were twofold: 1) To propose a novel subspace projection approach to quantify respiratory variation in the f-wave frequency trend that is robust to noise and can handle time-varying respiration, and 2) to investigate the impacts of deep breathing on the f-wave frequency in a population of AF patients without a pacemaker.

## 2 Materials and methods

**FIGURE 1:**
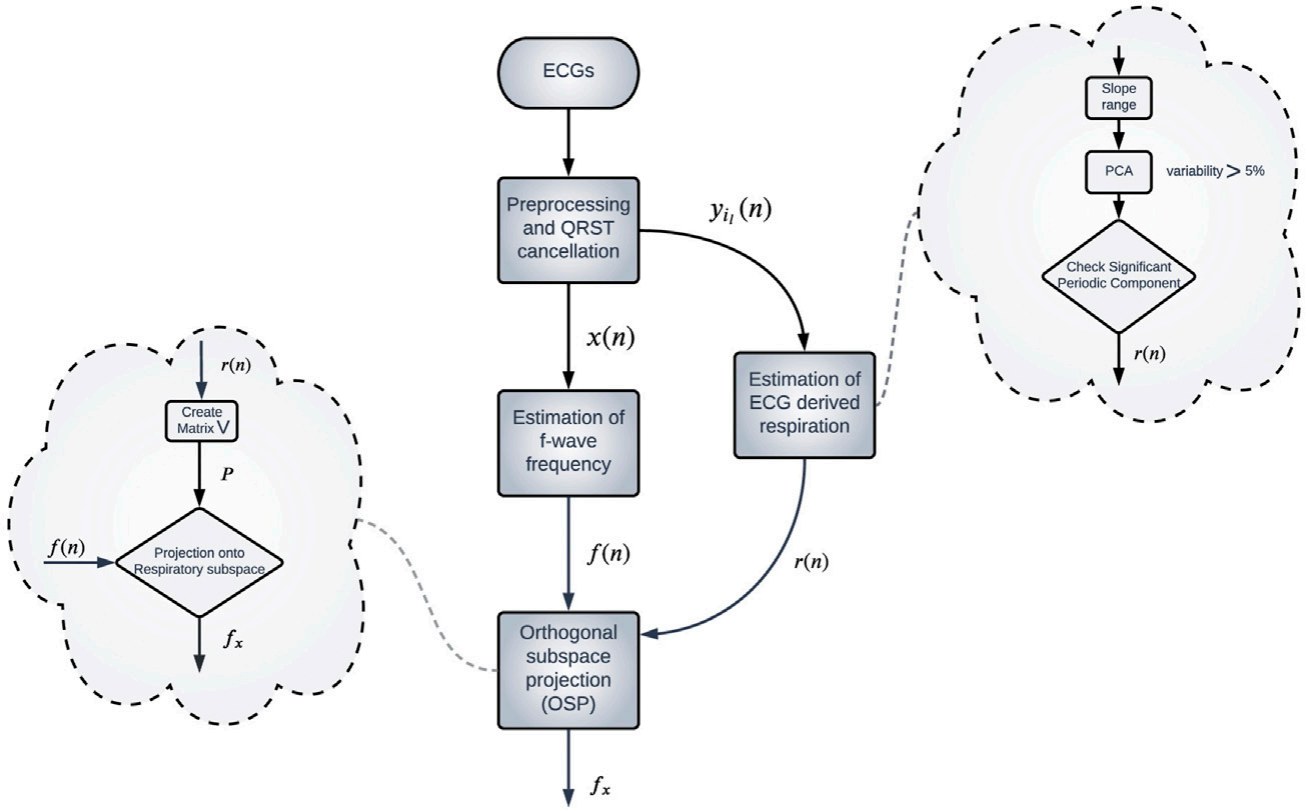
Schematic representation of the methodology.

A schematic overview of the methodology is shown in Figure 1. The clinical data is described in Section 2.1, the ECG processing aiming to obtain an f-wave signal *x*(*n*) is explained in Section 2.2. As follow, a model-based approach is applied to the extracted signal *x*(*n*) to estimate an f-wave frequency trend *f*(*n*) (Section 2.3). An ECG-derived respiration signal *r*(*n*) is estimated using the slope range approach (Section 2.4). Respiratory variation in *f*(*n*) is estimated using orthogonal subspace projection method (Section 2.5). Simulated f-wave signals are used to evaluate the performance of the proposed methodology (Section 2.6). Finally, statistical tests are applied to the results from analysis of clinical data to determine if there is a significant differences in heart rate, f-wave frequency, and respiratory variation in f-wave frequency trend between deep breathing phase and baseline (Section 2.7).

### 2.1 ECG data

The study population consists of a subset of 28 participants from the Swedish cardiopulmonary bioimage study (SCAPIS) (Bergström et al., 2015) that were diagnosed with AF. The clinical characteristics of the study population are summarized in Table 1. The subjects performed a deep breathing task. The task was 5-s inhalation and 5-s exhalation as deep-breathing (D) phase for 1 min, and as follows 5 min during baseline (B); the patients were in AF during the recordings. For further details on the study protocol, the reader is referred to (Engström et al., 2022). A standard 12-lead ECG at 500 Hz sampling rate was recorded throughout the protocol.

**TABLE 1:** Clinical characteristics of patients.

	Number
Age (*mean* ± SD, range)	60.1 ± 4.0 [50.1–64.9]
Men (%)	23 (82)
BMI (*mean* ± SD, range)	31.8 ± 7.2 [18.8–50.8]
Systolic BP	124 ± 23 [90–188]
Diastolic BP	79.9 ± 11 [61–104]
Hypertension*(%)	17 (61)
Diabetes (%)	2 (7)
Never smokers (%)	9 (32)
Heart failure (%)	2 (7)
Previous AMI or angina (%)	2 (7)
Treatment	
Beta blocker (%)	15 (54)
Ca-antagonist (%)	6 (21)
Antiarrhythmic drug (%)	4 (14)

∗ ≥140/90 mmHg or treatment for hypertension.

### 2.2 ECG processing

The CardioLund ECG parser (CardioLund Research AB, Lund, Sweden) is used for preprocessing, beat-detection, and beat classification and QRST cancellation. Briefly, in this software, a linear-phase high-pass filter is applied to the ECG to eliminate baseline wander, and fiducial points in the QRS complexes are detected; also, the QRS complexes are classified based on their morphology. The ectopic beats were identified based on correlation to template beats and were clustered and treated as a separate class for the QRST-cancellation. The QRS interval $y_{i_{l}}\left(n\right)$ is set to 140 ms, starting 110 ms before the end of the S wave and finishing 30 ms after the end of the S wave, where *i* and *l* denotes beat-number and lead, respectively. A spatiotemporal QRST cancellation approach (Stridh and Sörnmo, 2001) is employed to extract f-wave signals from the ECG. This average beat subtraction method compensates for minor morphological variations in the QRST complex by combining beat averages from different leads. For each beat class, one beat average is calculated and used for QRST cancellation in the corresponded beats in the ECG leads. The extracted f-wave signal is downsampled from 1 kHz to 50 Hz using appropriate low-pass filtering and decimation since such signals have negligible frequency content above 25 Hz. In the present study, the extracted f-wave signal from lead V1 denoted *x*(*n*), is subjected to analysis. For further analysis, the ECG data was divided into 1-min segments, resulting in five segments at baseline and one segment during deep breathing. The AFR and respiratory f-wave modulation was estimated from each 1-min segment of *x*(*n*) as described in Section 2.3 and Section 2.5, respectively. A respiration signal, which is required for estimation of respiratory f-wave modulation, was obtained from the corresponding QRS intervals $y_{i_{l}}\left(n\right)$ as described in Section 2.4; ectopic beats were removed for this analysis. For each patient, results from the 1-min segments recorded at baseline were averaged to obtain the heart rate (*HR* ^*B*^), atrial fibrillatory rate (*AFR* ^*B*^), and respiratory f-wave frequency modulation $\left(\Delta {f_{\mathrm{OSP}}}^{B}\right)$ , respectively. The corresponding estimates during deep breathing (*HR* ^*D*^, *AFR* ^*D*^, $\Delta {f_{\mathrm{OSP}}}^{D}$ ) were based on one segment.

### 2.3 Estimation of f-wave frequency trend

For the estimation of a high-resolution f-wave frequency trend, a harmonic f-wave model (Henriksson et al., 2018) is employed. The model f-wave signal is defined as the sum of a complex exponential signal with fundamental frequency *f* and its second harmonic,

$$s\left(n;\theta \right)=\overset{2}{\underset{m=1}{\sum }}\;A_{m}e^{j\left(m2\pi \frac{f}{f_{s}}n+\phi_{m}\right)},$$ (1)

where *A*_*m*_ and *ϕ*_*m*_ denote the amplitude and phase of *m*:th harmonic, respectively, and *f*_*s*_ is sampling frequency. The use of two harmonics in the model is motivated by the observations in (Henriksson et al., 2018), that additional harmonic results in more noise due to the additional degrees of freedom of this model. The parameters $\theta ={\left[f\;A_{1}\;A_{2}\;\phi_{1}\;\phi_{2}\right]}^{T}$ , are estimated by fitting the harmonic model *s* (*n*; ***θ***) to the analytic equivalent of *x*(*n*), denoted *x*_*a*_(*n*), using maximum likelihood approach.

$$\hat{\theta }=\underset{\theta }{\mathrm{arg min}}\|x_{a}\left(n\right)-s\left(n;\theta \right){\|}^{2},$$ (2)

The model is fitted to 20 ms overlapping 0.5-second segments of *x*_*a*_(*n*). For this fitting, *f* is constrained to the interval [*f*_0_ ± 1.5] Hz, where global frequency estimate (*f*_0_) is the maximum peak in the interval [4,12] Hz of the Welch periodogram of the whole *x*(*n*). The estimates of *f* result in an f-wave frequency trend *f*(*n*) sampled at 50 Hz. Then, correspond to the sampling rate of the respiration signal (cf. Section 2.4), *f*(*n*) is resampled to 5Hz. To quantify accuracy of the fitted model, a signal quality index, denoted S , is computed

$$S=1-\frac{\sigma_{\hat{e}}}{\sigma_{x_{a}}},$$ (3)

where $\sigma_{\hat{e}}$ and $\sigma_{x_{a}}$ denote the standard deviation of the model error $\left(\hat{e}\left(n\right)=x_{a}\left(n\right)-s\left(n;\hat{\theta }\right)\right)$ and *x*_*a*_(*n*), respectively. In this study, S is computed for non-overlappning 5 s segments. S ranges from 0 to 1, where a higher value corresponds to a better fit. Only segments with S>0.3 is considered for further analysis, since previous studies has shown that S larger than 0.3 was sufficient for accurate estimation of *f*(*n*) (Henriksson et al., 2018). The atrial fibrillatory rate (AFR) is estimated by the median of *f*(*n*).

### 2.4 Estimation of ECG-derived respiration

The slope range method (Kontaxis et al., 2019) is applied to each lead of the ECG separately to obtain a respiratory signal. The method quantifies variations in the QRS morphology, which are assumed to reflect respiratory activity, using the difference between the maximum and minimum derivative in the QRS interval,

$$r_{l}\left(i\right)=\underset{n}{\max}\left\{y_{i_{l}}^{\prime }\left(n\right)\right\}-\underset{n}{\min}\left\{y_{i_{l}}^{\prime }\left(n\right)\right\},$$ (4)

where *i* and *l* denotes beat-number and lead, respectively, and $y_{i_{l}}^{\prime }\left(n\right)=y_{i_{l}}\left(n\right)-y_{i_{l}}\left(n-1\right)$ . The resulting signal *r*_*l*_(*i*) is resampled to 5Hz using cubic spline interpolation to obtain a uniformly sampled signal *r*_*l*_(*n*). Principal component analysis (PCA) is applied to the set of *r*_*l*_(*n*) to derive a joint respiratory signal from all leads. The principal component that has the greatest variance and a significant periodic component in the respiratory interval ( $\left[\begin{array}{cc} 0.1 & 0.5 \end{array}\right]$ Hz) is selected as the respiratory signal, denoted as *r*(*n*). A principal component is considered to have a significant periodic component if the magnitude of the largest peak in the respiratory interval of its spectrum is at least 85% of the largest peak in the whole spectrum. The spectra are estimated by Welch periodograms based on 30 s sliding 25 s overlapping segments of PCA components. If none of the principal component accounting for more than 5% of the total variance has a significant periodic component in the respiration interval, no respiration signal is extracted.

### 2.5 Orthogonal subspace projection

To extract variations in the f-wave frequency trend that are linearly related to the respiration, an orthogonal subspace projection approach is employed (Chang, 2005). The demeaned *f*(*n*) denoted as $\tilde{f}\left(n\right)$ is projected onto a subspace defined by the matrix ***V***, constructed using the respiratory signal *r*(*n*),

$$V=\left[r_{0},r_{1},\ldots ,r_{d},\ldots ,r_{m}\right],$$ (5)

$$r_{d}={\left\{r\left(1+d\right),r\left(2+d\right),\ldots ,r\left(N-m+d\right)\right\}}^{T},$$ (6)

The model order *m* is determined by analysis of the simulated data (cf. Section 3.1). After creating the matrix ***V***, the signal $\tilde{f}\left(n\right)$ is projected onto the respiratory subspace using

$$f_{x}=V{\left(V^{T}V\right)}^{-1}V^{T}f=Pf,$$ (7)

where ***f*** is a length *N* vector of $\tilde{f}\left(n\right)$ , ***P*** is the projection matrix of size *N* − *m* × *N* − *m*, and ***f***_***x***_ is the component of ***f*** that is linearly related to respiration. The power of the variations linearly related to respiration $\left(f_{x}^{⊺}f_{x}\right)$ is a fraction of the total power of the variations (***f*** ^***⊺***^ ***f***). Assuming that the variations in ***f***_***x***_ are sinusoidal, the peak-to-peak amplitude is given by

$$\Delta {\bar{f}}_{\mathrm{OSP}}=\sqrt{\frac{2\cdot \left(f_{x}^{⊺}f_{x}\right)}{N}},$$ (8)

In the present study, $\Delta {\bar{f}}_{\mathrm{OSP}}$ serves as an estimate of the magnitude of the respiratory induced f-wave frequency variations.

### 2.6 Performance evaluation

Simulated f-wave signals were used in order to assess the performance of the orthogonal subspace projection approach and its dependence on model order *m* (Section 2.5), signal quality S (Section 2.3) and characteristics of the f-wave signals. The f-wave signals were simulated by a modified version of the saw-tooth model proposed by (Stridh and Sörnmo, 2001). The f-wave signal is the sum of a sinusoid and its harmonic with time-varying frequency

$$x_{\mathrm{sim}}\left(n\right)=\overset{2}{\underset{k=1}{\sum }}A_{k}\left(n\right)\sin\left(2\pi kF\left(n\right)n\right)+v\left(n\right),$$ (9)

$$F\left(n\right)=\frac{F}{F_{s}}+\frac{\Delta F}{2\pi F_{r}n}\sin\left(2\pi n\frac{F_{r}}{F_{s}}\right)+\frac{\Phi \left(n\right)}{2\pi kn},$$ (10)

where *F* defines the average fundamental frequency, and respiratory f-wave frequency variation is quantified by *F*_*r*_ and Δ*F*, defining the variation frequency and the variation magnitude, respectively. To incorporate other forms of variation in the f-wave frequency, random phase variation, Φ(*n*), is added; it is modeled as white Gaussian noise with standard deviation *σ*_Φ_. The amplitude of the *k*:th harmonic is given by

$$A_{k}\left(n\right)=\frac{2}{k\pi }\left(A+\Delta A\left(n\right)\right),$$ (11)

where *A* is the average f-wave amplitude, and Δ*A*(*n*) quantifies random amplitude variation and is assumed to have a Gaussian distribution with mean zero and standard deviation *A*/5; the parameter *A* was chosen to obtain a signal standard deviation of signal *σ*_*x*_ equal to 50. The following parameters were used for simulating one minute-long f-wave signals: *F* = {4, 5, 6, 7, 8, 9, 10} Hz, *F*_*r*_ = {0.1, 0.15, 0.20, 0.25, 0.30} Hz, Δ*F* = {0, 0.025, 0.05, … , 0.3} Hz, *σ*_Φ_ = {0.27, 0.40, 0.55, 0.67, 0.80}. White Gaussian noise *v*(*n*) with *σ*_*v*_ = {0.1*A*, 0.2*A*, 0.3*A*, 0.4*A*, 0.5*A*} is added to form realistic f-wave signals and the sampling frequency was set to *F*_*s*_ = 50 Hz. Ten realizations of *x*_*sim*_(*n*) for each parameter setting were considered, resulting in a total of 113,750 simulated signals.

Through these simulated signals, the accuracy of $\Delta {\bar{f}}_{\mathrm{OSP}}$ as an estimate of Δ*F* is compared to our previously proposed band-pass filtering approach to quantify respiratory induced variations in the f-wave frequency trend (Abdollahpur et al., 2021). In that method, respiratory variation is estimated by applying a narrow band-pass filter with a fixed bandwidth of 0.06 Hz and a center frequency corresponding to the *F*_*r*_. The f-wave frequency trend *f*(*n*) obtained as described in Section 2.3. The average envelope of the filtered *f*(*n*), denoted $\Delta {\bar{f}}_{BP}$ , is used to quantify the magnitude of the respiratory variation. The absolute difference between Δ*F* and $\Delta {\bar{f}}_{BP}$ , denoted as *ϵ*_*BP*_, and the absolute difference between Δ*F* and $\Delta {\bar{f}}_{\mathrm{OSP}}$ denoted as *ϵ*_*OSP*_ are used to assess the performance of the methods.

### 2.7 Statistical analysis

Results are presented as mean ± std, and as median (range) for Gaussian and non-gaussian variables, respectively; the Lilliefors test is used to test for gaussianity. Student’s t-test and Wilcoxon signed-rank test are applied to determine if differences are significant for Gaussian and non-gaussian variables, respectively. Hence, a paired *t*-test is applied to evaluate the difference between *ϵ*_*OSP*_ and *ϵ*_*BP*_, and a Wilcoxon signed-rank test is applied to determine whether differences in *HR*, *AFR*, and Δ*f*_*OSP*_ between baseline and deep breathing are significant. Further, Spearman rank correlation is used to evaluate the relationship between changes in *HR*, *AFR*, and Δ*f*_*OSP*_ in response to deep breathing. The level of statistical significance is considered *p* < 0.05.

## 3 Results

### 3.1 Simulations

**FIGURE 2:**
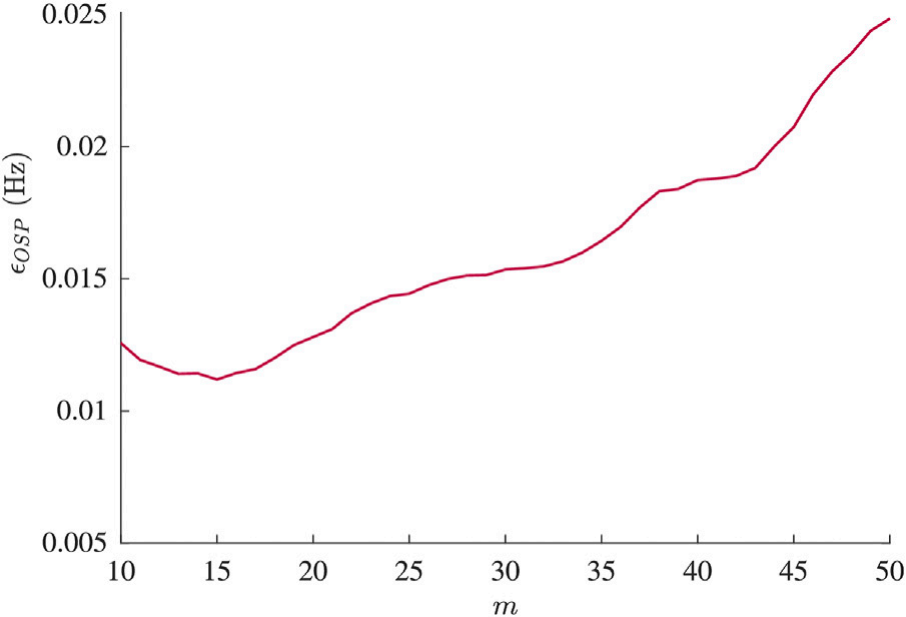
Mean estimation error *ϵ*_*OSP*_ from simulation plotted versus model order *m*.

**FIGURE 3:**
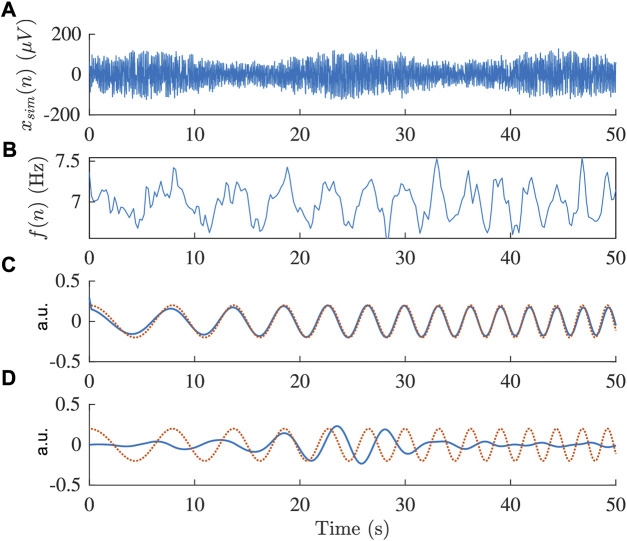
**(A)** Simulated signal *x*_*sim*_(*n*) and **(B)** corresponding estimated frequency trend *f*(*n*), respectively. **(C,D)** Modeled respiration signal (red) and extracted respiratory component (blue) obtained using **(C)** orthogonal subspace projection and **(D)** bandpass filtering.

**FIGURE 4:**
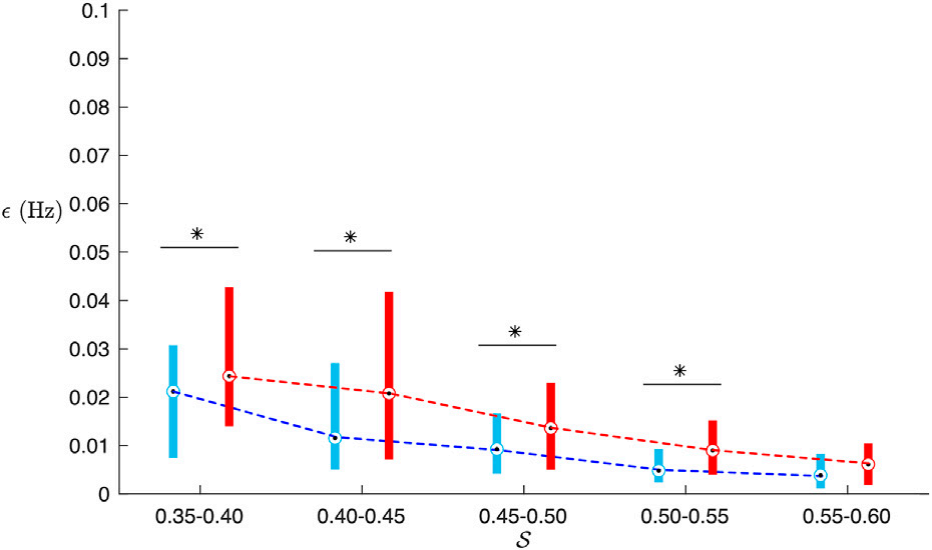
Box-plot of *ϵ*_*BP*_ (red) and *ϵ*_*OSP*_ (blue) from simulation as a function of signal quality S . (∗) denotes significant differences (*p* <0.05).

**FIGURE 5:**
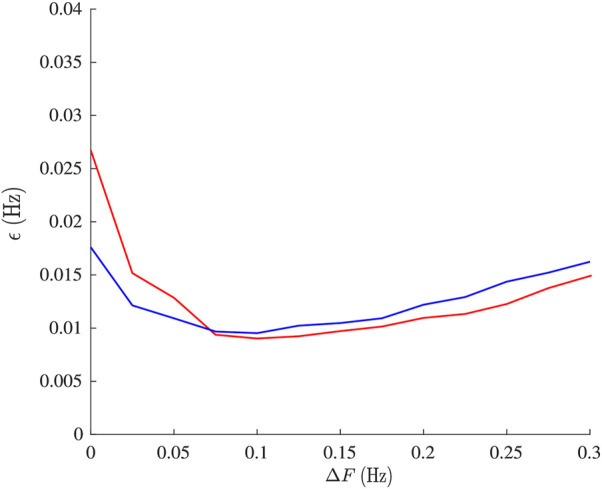
Mean estimation error *ϵ*_*OSP*_ (red) and *ϵ*_*BP*_ (blue) from simulations plotted versus the magnitude of respiratory modulation Δ*F*.

Results from the analysis of simulated data are presented in Figures 2–5. From Figure 2, it is apparent that the smallest *ϵ*_*OSP*_ was achieved for *m* = 15, and hence, *m* was set to 15 for the remaining analysis. The effect of the time-varying respiration is illustrated in Figure 3 where *F*_*r*_ changes from 0.1 to 0.3 Hz during 1 minute. As shown in Figure 3, the respiratory variations can be accurately extracted using the orthogonal subspace projection approach, while the previously proposed bandpass filtering approach fails. The *ϵ*_*OSP*_ was significantly smaller than *ϵ*_*BP*_ (0.017 ± 0.012 vs. 0.021 ± 0.015, *p* < 0.001). The improved accuracy obtained with the orthogonal subspace projection approach is more prominent for lower values of S, corresponding to higher noise levels, cf. Figure 4. The accuracy of the estimates was not affected by the f-wave frequency and the respiration rate (results not shown). However, for both approaches the estimates were less accurate for respiratory variations of small magnitudes (Δ*f* < 0.075 Hz), cf. Figure 5.

### 3.2 Heart rate and f-wave frequency

**FIGURE 6:**
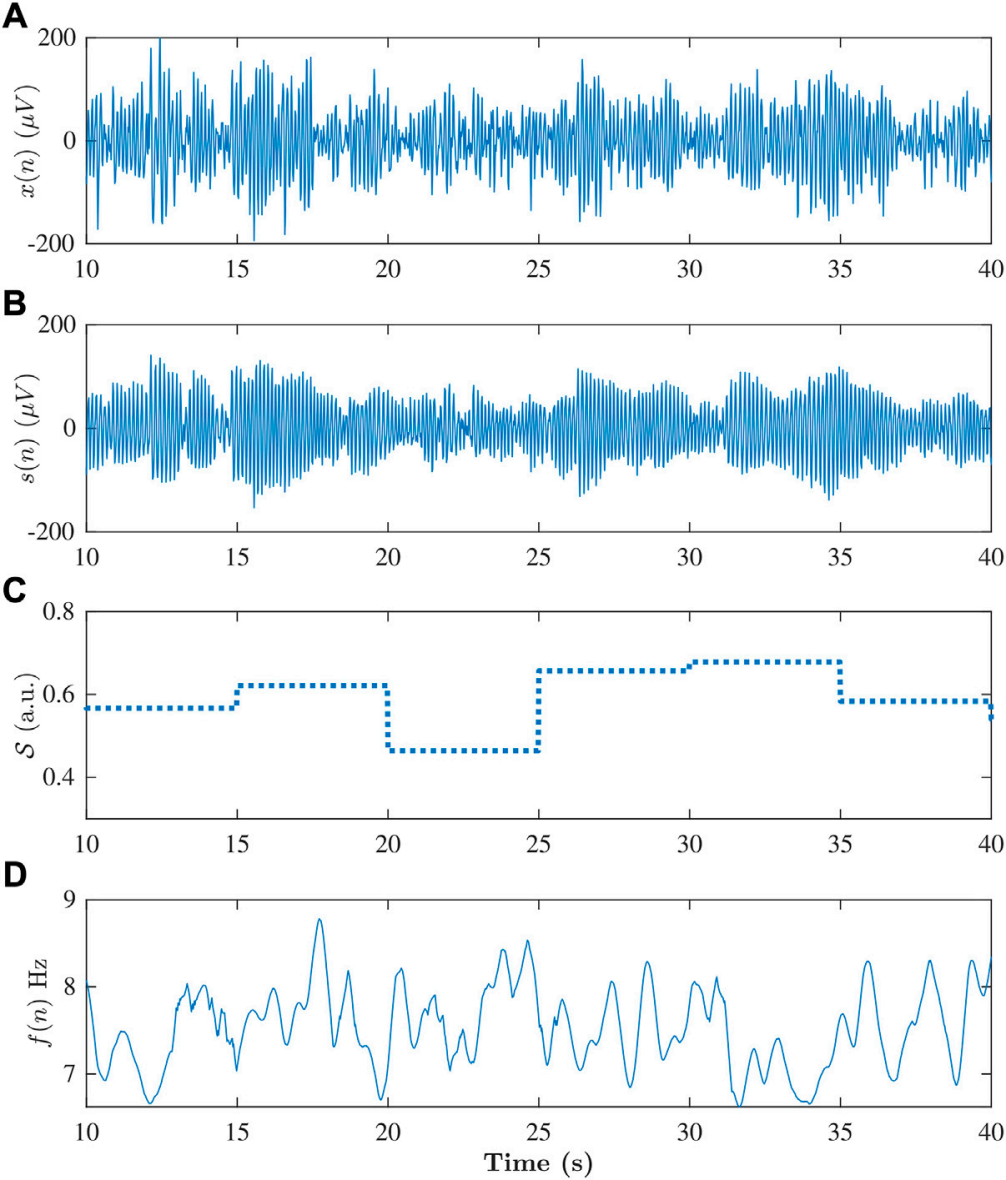
**(A)** Extracted f-wave signal *x*(*n*), and **(B)** corresponding modeled signal *s*(*n*), **(C)** signal quality index S and **(D)** extracted f-wave frequency trend *f*(*n*) obtained from a 30 s ECG segment from one of the patient at baseline.

**FIGURE 7:**
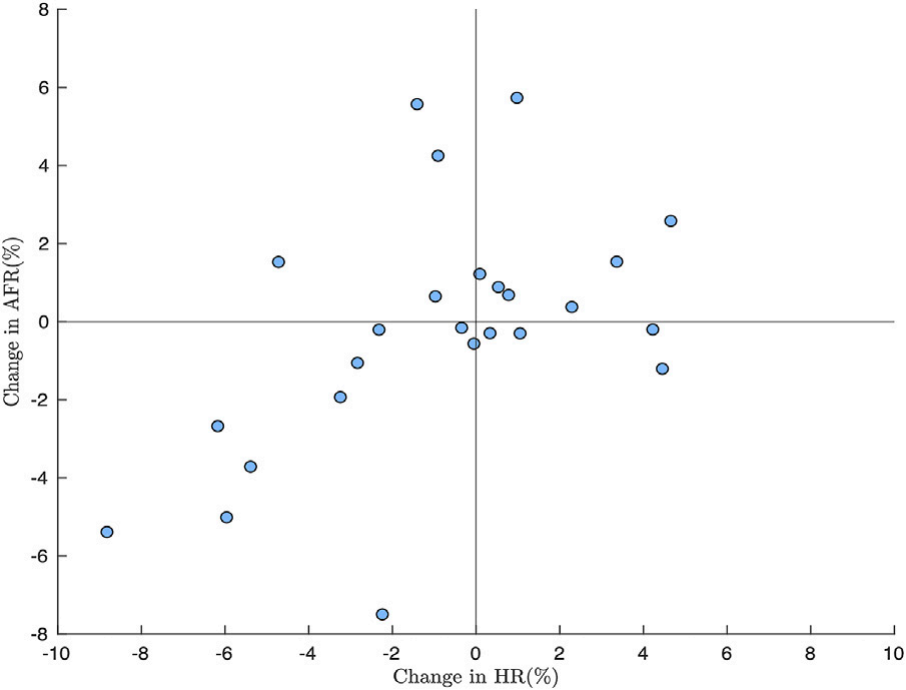
Relative changes between *AFR* ^*D*^ and *AFR* ^*B*^ plotted versus relative between *HR* ^*D*^ and *HR* ^*B*^

An example of a 30-second f-wave signal *x*(*n*) and the corresponding model signal *s* (*n*; ***θ***), signal quality index S , and extracted f-wave frequency trend *f*(*n*) is displayed in Figure 6. The signal quality was sufficient (S>0.3) for estimation of *f*(*n*) in 98% of the data. The *AFR* ^*B*^ was 6.93 (4.65–6.97) Hz and *AFR* ^*D*^ was 6.94 (4.56–6.99) Hz, a paired *t*-test showed no significant difference between baseline and deep breathing. The *HR* ^*B*^ was 75.5 (37–150) bpm and *HR* ^*D*^ was 74 (37–146) bpm; there were no significant differences between baseline and deep breathing. The changes in AFR versus changes in HR are displayed in Figure 7. The changes in *AFR* range between −8 and 6%, and the changes in *HR* range between −9 and 5%. There was a weak correlation between changes in HR and changes in AFR (*r* = 0.38, *p* < 0.03). The linear dependence between changes in HR and changes in AFR appears to be more pronounced for patients where the heart rate decreases in response to deep breathing, cf. Figure 7.

### 3.3 Respiration

**FIGURE 8:**
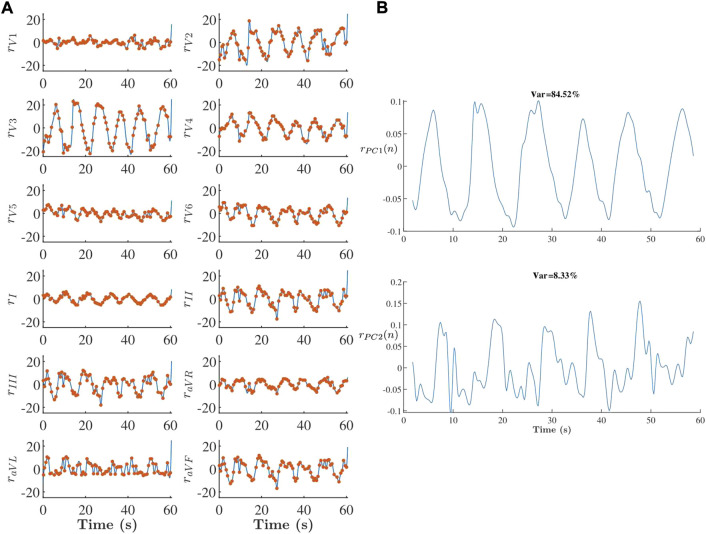
**(A)** Respiration signals *r*_*l*_(*n*) (blue line) and *r*_*l*_(*i*) (red dots) derived from 1-min ECG during deep breathing and **(B)** corresponding PCA components. In this example *r*(*n*) is set to $r_{PC_{1}}\left(n\right)$ since it has a significant periodic component and accounts for most of the total variance.

Figure 8 gives an example of extracted respiratory signals *r*_*l*_(*n*) and the corresponding principal components for one patient during deep breathing. In this example, both $r_{PC_{1}}\left(n\right)$ and $r_{PC_{2}}\left(n\right)$ have a significant periodic component according to definition in Section 2.4. The $r_{PC_{1}}\left(n\right)$ is selected as respiration signal (*r*(*n*)) since it has the largest variance. Respiration signals *r*(*n*) could be obtained from 118 out of 168 (70%) of the analyzed 1-min segments. The estimated respiration rate *F*_*r*_ was significantly higher at baseline (0.20 ± 0.06) Hz than during deep breathing (0.10 ± 0.01) Hz; the *F*_*r*_ estimated during deep breathing corresponded to the respiration frequency in controlled of the study protocol.

### 3.4 Respiratory f-wave variation

**FIGURE 9:**
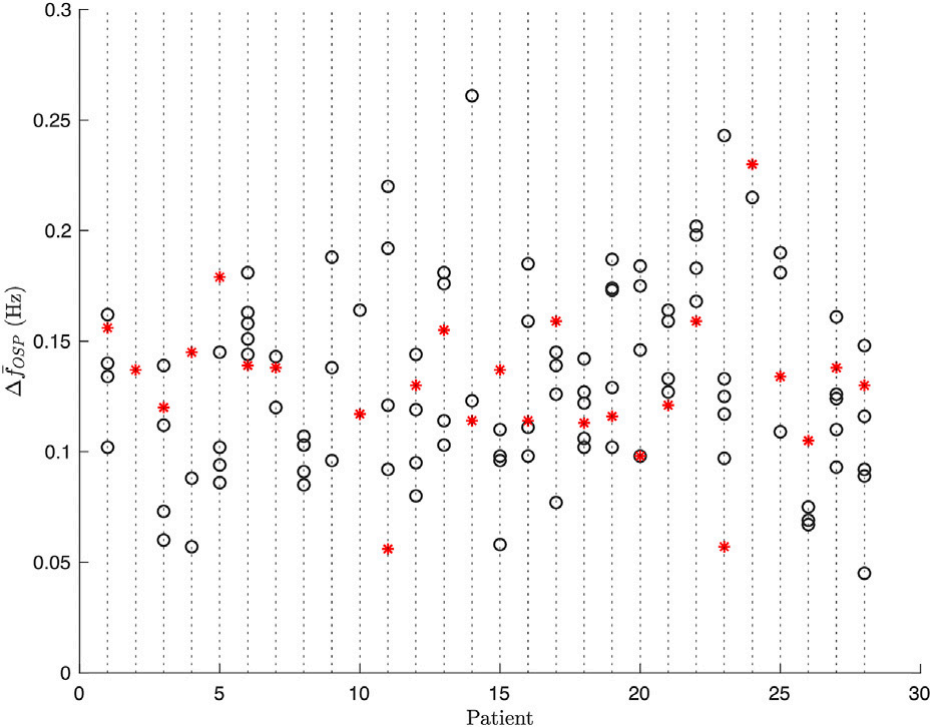
Estimates of respiratory f-wave frequency variations $\Delta {f_{\mathrm{OSP}}}^{B}$ (black circle) and $\Delta {f_{\mathrm{OSP}}}^{D}$ (red ∗) from all 1-min segments for each patient.

**FIGURE 10:**
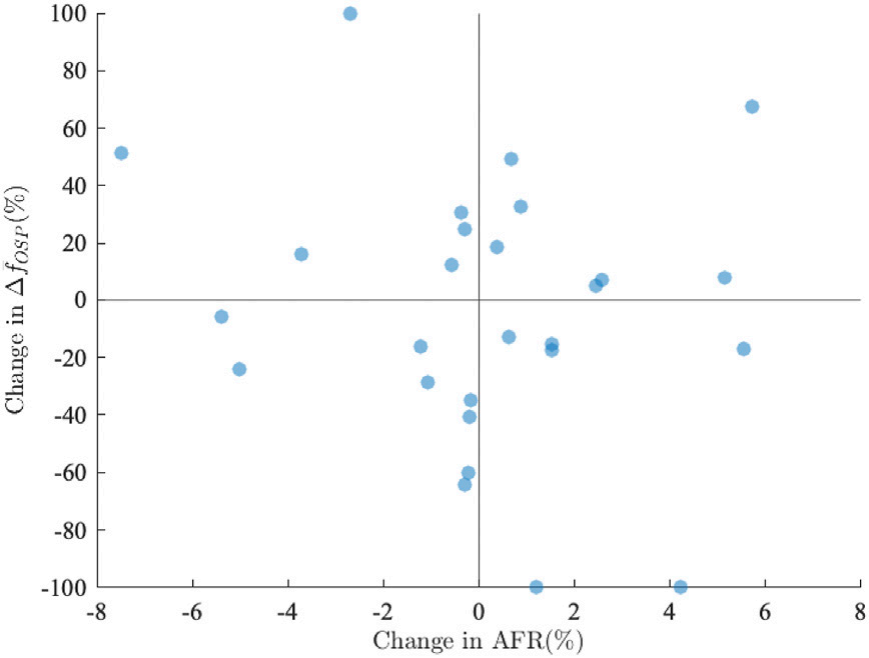
Relative change between $\Delta {f_{\mathrm{OSP}}}^{B}$ and $\Delta {f_{\mathrm{OSP}}}^{D}$ plotted versus relative change between *AFR* ^*B*^ and *AFR* ^*D*^.

The $\Delta {\bar{f}}_{\mathrm{OSP}}$ could be obtained from all 1-min segments with a valid respiration signal, i.e., 118 out of 168.; these estimates are displayed in Figure 9. The $\Delta {f_{\mathrm{OSP}}}^{B}$ was 0.130 (0.045–0.260) Hz and $\Delta {f_{\mathrm{OSP}}}^{D}$ was 0.130 (0.056–0.230) Hz. A paired *t*-test showed no significant differences between baseline and deep breathing. The changes in $\Delta {\bar{f}}_{\mathrm{OSP}}$ from phase from baseline to deep breathing plotted versus the corresponding changes in the *AFR* in Figure 10. The changes ranged from −100 to 100% for $\Delta {\bar{f}}_{\mathrm{OSP}}$ . The Spearman method showed no correlation between changes in $\Delta {\bar{f}}_{\mathrm{OSP}}$ and changes in the *AFR*.

## 4 Discussion

In the present study, we propose a novel methodology, based on orthogonal subspace projection, for quantifying respiratory variations in the f-wave frequency trend. Results from analysis of simulated data show that the estimation accuracy of the proposed approach is comparable to that of our previously proposed bandpass filtering approach (Abdollahpur et al., 2021). However, the proposed approach is better suited for analysis of standard ECG recordings since it can handle time-varying respiration (Figure 3) and provides more accurate estimates of respiratory variations at low SNR (Figure 4).

Orthogonal subspace projection has previously been proposed for removing respiratory influences in heart rate variability signal for improved estimation of sympathovagal balance (Varon et al., 2018). In contrast, in this study we aimed to keep variations in f-wave frequency trend that were linearly related to respiration and remove other variations. Such respiratory–induced f-wave frequency variations have previously been shown to be affected by parasympatetic regulation in a cohort of AF patients with complete AV block and pacemaker set at a fixed pacing rate (Abdollahpur et al., 2021).

The orthogonal subspace projection relies on an ECG-derived respiration (EDR) signal. It should be noted that standard RR interval-based algorithms for ECG-derived respiration (Widjaja et al., 2014) are not applicable during AF since variations in heart rate during AF do not originate from the sinus node. Hence, the slope range method was selected for this since previous studies (Kontaxis et al., 2019) has shown that this method is robust during AF and provides more accurate estimates compared to QRS loop rotation angle (Bailón et al., 2006) and the R-wave angle (Lázaro et al., 2014). In the slope range method f-wave suppression is not needed and its performance is less affected by the presence of f-waves (Kontaxis et al., 2019). An EDR signal is derived from each lead separately, and PCA is employed to merge respiratory information from EDR signals from different leads. It is assumed that the PCA component that has a significant periodicity in the 0.1–0.4 Hz interval and accounts for the largest part of the variations in the EDR signals contains the respiratory information. Respiration signals couldn’t be obtained from 30% of the analyzed 1-min segments due to criteria defined in Section 2.4. Since this methodology aimed for the effect of time-varying respiration on the f-wave frequency, we used PCA to find respiration signal. The PCA uses a maximum-variance criteria to separate respiration signal and noise into orthogonal subspaces. Its components are sensitive to the high variance noise, which may not be the best way to find respiration signals. An alternative solution would be to use periodic component analysis (Saul and Allen, 2000), which has previously been proposed for decomposition of multilead ECG (Sameni et al., 2008) and applied for analysis of, e.g., t-wave alternans (Monasterio et al., 2010; Palmieri et al., 2021). Whereas PCA uses a maximum-variance criteria to decompose signals, periodic component analysis maximizes the periodic structure. Periodic component analysis has the advantage of being less sensitive to large amplitude noise, however, it is requires prior knowledge on the periodicity of the desired signal.

Respiratory induced f-wave frequency variations has previously been shown to be affected by parasympathetic regulation. In our previous study, (Abdollahpur et al., 2021), 5-min ECGs recorded from eight patients during controlled respiration before and after full vagal blockade were analyzed; in 50% of the patients, respiratory variation was significantly reduced after the vagal blockade. Moreover, results from computational simulations of human atrial tissues confirmed that the pattern of the parasympathetic neurotransmitter acetylcholine release could be an important factor involved in f-wave frequency variation (Celotto et al., 2020). These results suggest that respiratory f-wave frequency variations can potentially be used to quantify ANS activity, which is of clinical interest since ANS activity is an important factor on the maintenance and progression of AF (Linz et al., 2019). For example, vagus nerve stimulation has been shown to shorten the atrial effective refractory period and suppress autonomic remodeling in dogs with obstructive apnea induced AF (Yu et al., 2017). Further, it has been shown that AF progression through cellular remodeling could be reduced by minimizing sympathetic or increasing parasympathetic tone (Bashir et al., 2019). In a recent study (Sohinki et al., 2021), investigated the impact of low-level electromagnetic fields (LL-EMF) which is specifically targeted for vagal stimulation, on AF inducibility in humans.

In present study no significant differences were found between f-wave frequency variations at baseline and during deep breathing. Several factors could contribute to this observation. Firstly, the duration of the deep breathing task was just 1 min which may not be sufficient time to observe the effect of changes in autonomic tone on the f-wave frequency trend. Further, considering the large variation of $\Delta {\bar{f}}_{\mathrm{OSP}}$ from the 1-min segments at baseline (cf Figure 9), recordings of longer duration during deep breathing are desired for robust estimation. Secondly, the heterogeneous behavior of changes in $\Delta {\bar{f}}_{\mathrm{OSP}}$ in response to deep breathing may be due to individual differences in AF progression which may effect the ANS regulation (Linz et al., 2019). The patients in the present study have paroxysmal and persistent AF with unknown duration. However, due to the small study population subgroup analysis is not possible. Thirdly, the fluctuations in intrathoracic pressure as a result of respiration have an important effect on the heart rate during normal sinus rhythm. The effect of these fluctuations on the heart rate during AF are largely unknown. It is possible that variations in heart rate counteract the impact of respiration on the fluctuations in acetylcholine level in the atrial tissues and, as a result, the f-wave frequency variation. In the previous study, the effect of the parasympathetic activity was investigated in a cohort of AF patients with complete AV block and fixed-rate (60 beat/min) pacemaker (Abdollahpur et al., 2021) and hence the effect of changes in ANS activity induced by variations in heart rate was eliminated. In contrast, the present dataset consists of patients without a pacemaker. Finally, it should be noted that the estimation accuracy of the proposed methodology sets a lower bound for changes that can be detected (cf. Section 3.1), and we cannot exclude the possibility that there are changes below this limit that remain undetected.

## 5 Conclusion

We propose a novel orthogonal subspace projection approach to quantify respiratory variations in the f-wave frequency trend obtained from the ECG during AF. Results from simulated f-wave signals show that the proposed approach offers more robust performance in respiratory variation estimation compared to the previously proposed bandpass filtering approach. Results from analysis of clinical data were heterogeneous and no significant differences in HR, AFR and respiratory f-wave frequency variations $\Delta {\bar{f}}_{\mathrm{OSP}}$ between baseline and deep breathing were found in SCAPIS dataset.

## Data Availability

The data analyzed in this study is subject to the following licenses/restrictions: The data is owned by Lund University. Requests to access these datasets should be directed to the steering committee of SCAPIS. Requests to access these datasets should be directed to scapis@scapis.org.

## References

[B1] Abdollahpur M. Holmqvist F. Platonov P. Sandberg F. (2021). Respiratory induced modulation in f-wave characteristics during atrial fibrillation. Front. Physiol. 12, 653492. 10.3389/fphys.2021.653492 33897462 PMC8060635

[B2] Bailón R. Sornmo L. Laguna P. (2006). A robust method for ecg-based estimation of the respiratory frequency during stress testing. IEEE Trans. Biomed. Eng. 53, 1273–1285. 10.1109/TBME.2006.871888 16830932

[B3] Bashir M. U. Bhagra A. Kapa S. McLeod C. J. (2019). Modulation of the autonomic nervous system through mind and body practices as a treatment for atrial fibrillation. Rev. Cardiovasc. Med. 20, 129–137. 10.31083/j.rcm.2019.03.517 31601087

[B4] Bergström G. Berglund G. Blomberg A. Brandberg J. Engström G. Engvall J. (2015). The Swedish cardiopulmonary bioimage study: Objectives and design. J. Intern. Med. 278, 645–659. 10.1111/joim.12384 26096600 PMC4744991

[B5] Capucci A. Biffi M. Boriani G. Ravelli F. Nollo G. Sabbatani P. (1995). Dynamic electrophysiological behavior of human atria during paroxysmal atrial fibrillation. Circulation 92, 1193–1202. 10.1161/01.cir.92.5.1193 7648665

[B6] Celotto C. Sánchez C. Mountris K. A. Abdollahpur M. Sandberg F. Laguna P. (2020). Relationship between atrial oscillatory acetylcholine release pattern and f-wave frequency modulation: A computational and experimental study. Comput. Cardiol., 1–4.

[B7] Chang C.-I. (2005). Orthogonal subspace projection (osp) revisited: A comprehensive study and analysis. IEEE Trans. Geosci. Remote Sens. 43, 502–518. 10.1109/tgrs.2004.839543

[B8] Crandall M. A. Bradley D. J. Packer D. L. Asirvatham S. J. (2009). Contemporary management of atrial fibrillation: Update on anticoagulation and invasive management strategies. Mayo Clin. Proc. 84, 643–662. 19567719 10.1016/S0025-6196(11)60754-4 PMC2704137

[B9] Engström G. Hamrefors V. Fedorowski A. Persson A. Johansson M. E. Ostenfeld E. (2022). Cardiovagal function measured by the deep breathing test: Relationships with coronary atherosclerosis. J. Am. Heart Assoc. 11, e024053. 10.1161/JAHA.121.024053 35352566 PMC9075454

[B10] Fabritz L. Guasch E. Antoniades C. Bardinet I. Benninger G. Betts T. R. (2016). Expert consensus document: Defining the major health modifiers causing atrial fibrillation: A roadmap to underpin personalized prevention and treatment. Nat. Rev. Cardiol. 13, 230–237. 10.1038/nrcardio.2015.194 26701216

[B11] Henriksson M. Petrenas A. Marozas V. Sandberg F. Sornmo L. (2018). Model-based assessment of f-wave signal quality in patients with atrial fibrillation. IEEE Trans. Biomed. Eng. 65, 2600–2611. 10.1109/TBME.2018.2810508 29993509

[B12] Holmqvist F. Stridh M. Waktare J. E. P. Brandt J. Sörnmo L. Roijer A. (2005). Rapid fluctuations in atrial fibrillatory electrophysiology detected during controlled respiration. Am. J. Physiol. Heart Circ. Physiol. 289, H754–H760. 10.1152/ajpheart.00075.2005 16014618

[B13] Kontaxis S. Lázaro J. Corino V. D. Sandberg F. Bailón R. Laguna P. (2019). Ecg-derived respiratory rate in atrial fibrillation. IEEE Trans. Biomed. Eng. 67, 905–914. 10.1109/TBME.2019.2923587 31226064

[B14] Krijthe B. P. Kunst A. Benjamin E. J. Lip G. Y. Franco O. H. Hofman A. (2013). Projections on the number of individuals with atrial fibrillation in the European Union, from 2000 to 2060. Eur. Heart J. 34, 2746–2751. 10.1093/eurheartj/eht280 23900699 PMC3858024

[B15] Lázaro J. Alcaine A. Romero D. Gil E. Laguna P. Pueyo E. (2014). Electrocardiogram derived respiratory rate from qrs slopes and r-wave angle. Ann. Biomed. Eng. 42, 2072–2083. 10.1007/s10439-014-1073-x 25118665

[B16] Linz D. Elliott A. D. Hohl M. Malik V. Schotten U. Dobrev D. (2019). Role of autonomic nervous system in atrial fibrillation. Int. J. Cardiol. 287, 181–188. 10.1016/j.ijcard.2018.11.091 30497894

[B17] Lip G. Y. Nieuwlaat R. Pisters R. Lane D. A. Crijns H. J. (2010). Refining clinical risk stratification for predicting stroke and thromboembolism in atrial fibrillation using a novel risk factor-based approach: The euro heart survey on atrial fibrillation. Chest 137, 263–272. 10.1378/chest.09-1584 19762550

[B18] Miyasaka Y. Barnes M. E. Gersh B. J. Cha S. S. Bailey K. R. Abhayaratna W. P. (2006). Secular trends in incidence of atrial fibrillation in olmsted county, Minnesota, 1980 to 2000, and implications on the projections for future prevalence. Circulation 114, 119–125. 10.1161/CIRCULATIONAHA.105.595140 16818816

[B19] Monasterio V. Clifford G. D. Laguna P. MARTInez J. P. (2010). A multilead scheme based on periodic component analysis for t-wave alternans analysis in the ecg. Ann. Biomed. Eng. 38, 2532–2541. 10.1007/s10439-010-0029-z 20387121

[B20] Nitta T. Ishii Y. Miyagi Y. Ohmori H. Sakamoto S.-i. Tanaka S. (2004). Concurrent multiple left atrial focal activations with fibrillatory conduction and right atrial focal or reentrant activation as the mechanism in atrial fibrillation. J. Thorac. Cardiovasc. Surg. 127, 770–778. 10.1016/j.jtcvs.2003.05.001 15001906

[B21] Östenson S. Corino V. D. A. Carlsson J. Platonov P. G. (2017). Autonomic influence on atrial fibrillatory process: Head-up and head-down tilting. Ann. Noninvasive Electrocardiol. 22, e12405. 10.1111/anec.12405 PMC6931695 27611110

[B22] Palmieri F. Gomis P. Ruiz J. E. Ferreira D. Martín-Yebra A. Pueyo E. (2021). Ecg-based monitoring of blood potassium concentration: Periodic versus principal component as lead transformation for biomarker robustness. Biomed. Signal Process. Control 68, 102719. 10.1016/j.bspc.2021.102719

[B23] Patel N. J. Deshmukh A. Pant S. Singh V. Patel N. Arora S. (2014). Contemporary trends of hospitalization for atrial fibrillation in the United States, 2000 through 2010: Implications for healthcare planning. Circulation 129, 2371–2379. 10.1161/CIRCULATIONAHA.114.008201 24842943

[B24] Platonov P. G. Corino V. D. Seifert M. Holmqvist F. Sörnmo L. (2014). Atrial fibrillatory rate in the clinical context: Natural course and prediction of intervention outcome. Europace 16, iv110–iv119. iv110–iv119. 10.1093/europace/euu249 25362161

[B25] Saksena S. Skadsberg N. D. Rao H. B. Filipecki A. (2005). Biatrial and three-dimensional mapping of spontaneous atrial arrhythmias in patients with refractory atrial fibrillation. J. Cardiovasc. Electrophysiol. 16, 494–504. 10.1111/j.1540-8167.2005.40531.x 15877620

[B26] Sameni R. Jutten C. Shamsollahi M. B. (2008). Multichannel electrocardiogram decomposition using periodic component analysis. IEEE Trans. Biomed. Eng. 55, 1935–1940. 10.1109/TBME.2008.919714 18632355

[B27] Saul L. Allen J. (2000). Periodic component analysis: An eigenvalue method for representing periodic structure in speech. Adv. Neural Inf. Process. Syst. 13.

[B28] Sohinki D. Thomas J. Scherlag B. Stavrakis S. Yousif A. Po S. (2021). Impact of low-level electromagnetic fields on the inducibility of atrial fibrillation in the electrophysiology laboratory. Heart Rhythm O2 2, 239–246. 10.1016/j.hroo.2021.04.004 34337574 PMC8322792

[B29] Stridh M. Meurling C. Olsson B. Sörnmo L. (2003). Detection of autonomic modulation in permanent atrial fibrillation. Med. Biol. Eng. Comput. 41, 625–629. 10.1007/bf02349969 14686587

[B30] Stridh M. Sörnmo L. (2001). Spatiotemporal QRST cancellation techniques for analysis of atrial fibrillation. IEEE Trans. Biomed. Eng. 48, 105–111. 10.1109/10.900266 11235581

[B31] Varon C. Lázaro J. Bolea J. Hernando A. Aguiló J. Gil E. (2018). Unconstrained estimation of hrv indices after removing respiratory influences from heart rate. IEEE J. Biomed. Health Inf. 23, 2386–2397. 10.1109/JBHI.2018.2884644 30507541

[B32] Waldo A. L. (2003). Mechanisms of atrial fibrillation. J. Cardiovasc. Electrophysiol. 14, S267–S274. 10.1046/j.1540-8167.2003.90401.x 15005213

[B33] Widjaja D. Caicedo A. Vlemincx E. Van Diest I. Van Huffel S. (2014). Separation of respiratory influences from the tachogram: A methodological evaluation. PloS one 9, e101713. 10.1371/journal.pone.0101713 25004139 PMC4086956

[B34] Yu L. Li X. Huang B. Zhou X. Wang M. Zhou L. (2017). Impacts of renal sympathetic activation on atrial fibrillation: The potential role of the autonomic cross talk between kidney and heart. J. Am. Heart Assoc. 6, e004716. 10.1161/JAHA.116.004716 28255078 PMC5524006

